# Denosumab-induced hypocalcemia post bariatric surgery—a severe and protracted course: a case report

**DOI:** 10.1186/s13256-023-03764-w

**Published:** 2023-03-02

**Authors:** Mohd Hazriq Awang, Sharifah Faradila Wan Muhamad Hatta, Aimi Fadilah Mohamad, Rohana Abdul Ghani

**Affiliations:** Faculty of Medicine, Universiti Teknologi MARA, Sungai Buloh, Malaysia

**Keywords:** Hypocalcemia, Bariatric surgery, Osteoporosis, Denosumab, Vitamin D deficiency

## Abstract

**Background:**

Denosumab is known to cause abnormalities in calcium homeostasis. Most of such cases have been described in patients with underlying chronic kidney disease or severe vitamin D deficiency. Previous bariatric surgery could also contribute to hypocalcemia and deterioration in bone health.

**Case presentation:**

We present a case of a 61-year-old Malay female with worsening bilateral limb weakness, paresthesia, and severe carpopedal spasm a week after receiving subcutaneous denosumab for osteoporosis. She had a history of gastric bypass surgery 20 years ago. Post gastric bypass surgery, she was advised and initiated on lifelong calcium, vitamin D, and iron supplementations that she unfortunately stopped taking 5 years after surgery. Her last serum blood tests, prior to initiation on denosumab, were conducted in a different center, and she was told that she had a low calcium level; hence, she was advised to restart her vitamin and mineral supplements. Laboratory workup revealed severe hypocalcemia (adjusted serum calcium of 1.33 mmol/L) and mild hypophosphatemia (0.65 mmol/L), with normal magnesium and renal function. Electrocardiogram showed a prolonged QTc interval. She required four bolus courses of intravenous calcium gluconate, and three courses of continuous infusions due to retractable severe hypocalcemia (total of 29 vials of 10 mL of 10% calcium gluconate intravenously). In view of her low vitamin D level of 33 nmol/L, she was initiated on a loading dose of cholecalciferol of 50,000 IU per week for 8 weeks. However, despite a loading dose of cholecalciferol, multiple bolus courses, and infusions of calcium gluconate, her serum calcium hovered around only 1.8 mmol/L. After 8 days of continuous intravenous infusions of calcium gluconate, high doses of calcitriol 1.5 μg twice daily, and 1 g calcium carbonate three times daily, her serum calcium stabilized at approximately 2.0 mmol/L. She remained on these high doses for over 2 months, before they were gradually titrated down to ensure sustainability of a safe calcium level.

**Conclusion:**

This case report highlights the importance of screening for risk factors for iatrogenic hypocalcemia and ensuring normal levels before initiating denosumab. The patient history of bariatric surgery could have worsened the hypocalcemia, resulting in a more severe presentation and protracted response to oral calcium and vitamin D supplementation.

## Introduction

Denosumab has already found its place among the important treatments of osteoporosis. The changes in bone metabolism, however, can affect calcium homeostasis and result in hypocalcemia. The incidence of denosumab-associated hypocalcemia has varied considerably between 1.5% and 69% in different patient populations [1]. The incidence was reported to be 42% in an end-stage renal failure cohort [2] and 12.4% in metastatic bone disease over 34 months [3].

In addition, bariatric surgery, with denosumab therapy, also carries a potential additional risk for hypocalcemia [4]. In this paper we would like to share our experience in managing a case of denosumab-induced severe hypocalcemia in a patient with a history of gastric bypass surgery.

## Case presentation

A 61-year-old Malay female presented to the emergency department with a 6 day history of bilateral lower limb weakness and numbness. This was associated with frequent carpopedal spasms of the hands and feet that had gradually worsened to the point where she was unable to use her hands, due to the severe muscle spasms on the day of admission. She had recently presented to a private center with a complaint of acute back pain. Thoracolumbar radiography showed evidence of L2 wedge fracture. She denied any history of a recent fall; hence, a diagnosis of fragility fracture was made and subcutaneous (SC) denosumab 60 mg was administered a week prior to her current presentation. She had history of gastric bypass surgery 20 years ago and bilateral total knee replacements in June 2021. She claimed that routine blood investigations during those admissions did not reveal any abnormalities. However, she had not had any bone mineral density (BMD) measurements done. Apart from that she does not have any other medical or surgical illness. As for her gynecological history, she had two full-term pregnancies with two live births and no miscarriages in the past. She attained menopause at the age of 50 years old and remained healthy. She is a full-time housewife and does not smoke, nor does she drink alcohol. There was no significant family history related to bone diseases.

On examination, her vital signs were stable with a pulse rate of 63 beats per minute, blood pressure of 123/57 mmHg, and temperature of 37.2 °C. Her height was 165 cm, with a weight of 80 kg and body mass index of 29.5 kg/m^2^. Chvostek and Trousseau signs were positive. Cardiovascular, respiratory, neurological, and gastrointestinal examinations were all unremarkable.

Initial laboratory investigations are presented in Table 1.

**Table 1:** Laboratory results at diagnosis

Blood investigations	Results	Normal values
Adjusted calcium	1.33 mmol/L	2.2–2.5
Phosphate	0.65 mmol/L	0.81–1.45
Alkaline phosphatase	265 U/L	35–105
Magnesium	0.95 mmol/L	0.66–0.99
Potassium	3.5 mmol/L	3.5–5.1
Urea	2.4 mmol/L	2.78–8.07
Creatinine	52 μmol/L	44–80
eGFR	> 90 mL/min/1.73 m^2^	> 60
Parathyroid hormone	34.6 pmol/L	1.96–8.49
25-OH Vit D (Vitamin D)	33 nmol/L	> 50

eGFR: Estimated Glomerular Filtration Rate

An electrocardiogram (ECG) revealed a prolonged QT interval. She received a bolus of 10 mL of 10% calcium gluconate twice in the emergency department, and was admitted to the ward for close monitoring with subsequent continuous intravenous calcium infusion. In view of her low 25-OH vitamin D level, she was initiated on a loading dose of 50,000 IU cholecalciferol weekly for 8 weeks. Apart from that, she also received a total of four 10 mL courses of 10% calcium gluconate boluses and three courses of continuous calcium gluconate infusion over 12–23 hours (total of 25 vials of 10 mL of 10% calcium gluconate). However, despite a loading dose of cholecalciferol, multiple courses of boluses, and infusions of calcium gluconate, her serum calcium hovered around only 1.8 mmol/L. After 8 days of repeated courses of intravenous calcium gluconate infusions, high doses of calcitriol 1.5 μg twice daily, and 1 g calcium carbonate three times daily, her serum calcium stabilized at approximately 2.0 mmol/L. Her medications on discharge included calcitriol 1.5 μg twice daily, calcium carbonate 1 g three times daily, and cholecalciferol of 50,000 IU weekly.

**Fig. 1:**
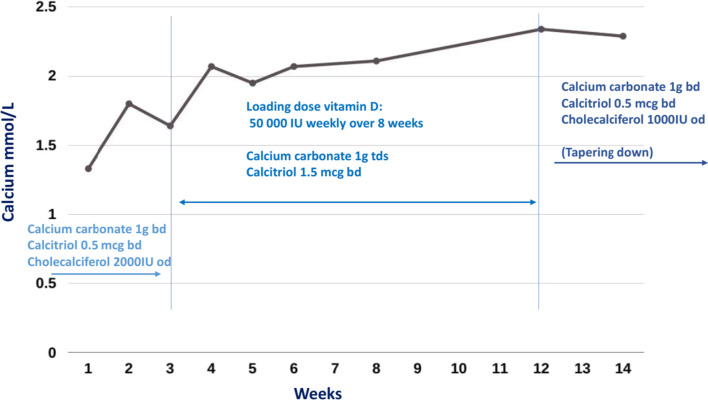
Changes in serum calcium and supplementation of calcium and vitamin D

The medications were only able to be reduced after 3 months to calcium carbonate 1 g twice daily and cholecalciferol of 1000 IU daily. This was able to be reduced further, and her calcium supplements were ceased after 6 months of treatment with continuation of cholecalciferol 1000 IU/day. At this point of time, her symptoms had completely resolved, and her serum calcium level remained stable at 2.36 mmol/L. Figure 1 demonstrates the delayed normalization of the serum calcium over the weeks after her presentation.

## Discussion

Denosumab, a drug administered via SC injection, is a human IgG_2(immunoglobulin)_ monoclonal antibody targeting the key bone resorption mediator receptor activator of nuclear factor kappa B ligand (RANKL). It was approved in 2010 to prevent skeletal-related events (SRE) in postmenopausal women with osteoporosis (Prolia), and in patients with solid tumor with bone metastasis (Xgeva) [5, 6]. The dose and frequency of administration of the drug is different for each indication. Patients with cancer will receive 120 mg once a month; meanwhile, those with osteoporosis receive doses that are approximately 10–12 times lower at 60 mg every 6 months. As denosumab is not cleared by the kidney, it does not require dose adjustments in those with chronic kidney disease [5].

In postmenopausal women, due to estrogen decline and in malignancies due to tumor-secreted growth factors, RANKL is upregulated. Denosumab works by binding to RANKL’s selectively with high affinity [7, 8], thus preventing RANKL from interacting and activating RANK (its receptor) on the surface of osteoclasts and their precursors. This leads to inhibition of formation, function, and survival of osteoclasts, resulting in reduced bone resorption [7].

It is generally well tolerated, with data in clinical trial studies of up to 10 years of treatment [9]. Adverse effects reported include atypical femoral fractures, osteonecrosis of the jaw, skin infections, dermatological adverse reactions, serious infections, and hypocalcemia [5, 6]. The incidence of denosumab-associated hypocalcemia reported in studies varied considerably in the range of 1.5–69% [1]. The reasons for this wide range include small sample sizes, short follow-up, and heterogeneity of the participants as trials included mixed populations of patients with osteoporosis, and patients with oncologic diseases [1]. Nonetheless, a randomized placebo-controlled study showed no significant differences in the number of symptomatic hypocalcemia cases in the denosumab arm, compared with the placebo [9], and differences were very rarely reported in its open-label, long-term follow-up study [9]. A recent, real-life study highlighted the importance of hypocalcemia in patients receiving denosumab with a prevalence of 7.4% among community-dwelling women with osteoporosis, albeit with mild symptoms [1].

Previous analyses of hypocalcemia in patients with cancer treated with denosumab have relied primarily on clinical adverse event reports, which mainly include symptomatic hypocalcemia events [10, 11]. Nonetheless, results from a meta-analysis of data from seven randomized controlled trials demonstrated a higher risk of adverse events of hypocalcemia among denosumab recipients, versus control groups [11]. In addition, hypocalcemia occurred more frequently in denosumab recipients than in zolendronic acid recipients, as measured by both laboratory values and adverse events [11]. This has led to the recommendation of monitoring calcium and mineral levels, specifically phosphorus and magnesium, within 14 days of denosumab injection [5, 6].

The pathophysiology of denosumab-associated hypocalcemia is believed to be due to the inactivation of osteoclasts, leading to a reduction in the release of calcium into the circulation [1]. It is, therefore, more prevalent among those with vitamin D deficiency, insufficient calcium intake, chronic kidney disease especially stages 4 and 5, hypoparathyroidism, high bone turnover, osteomalacia, rapid skeletal growth, Paget’s disease, hypomagnesemia, and prior bisphosphonate use [12]. Interestingly, in our patient, her risk factors for hypocalcemia would be low calcium and low vitamin D, prior to the administration of denosumab, most likely because of the bariatric surgery that she had undergone previously. Similar to a double hit theory, malabsorption from the gastric bypass surgery, combined with mechanical and hormonal factors related to weight loss, increased her likelihood of developing osteoporosis as well as vitamin and mineral deficiencies [13]. In addition, impaired absorption also poses a challenge in treatment; thus, these individuals may require higher doses of supplementations to ensure adequate levels of vitamins and minerals. Suboptimal preoperative nutrition, anatomical changes, and postoperative dietary constraints can lead to significant micronutrient and vitamin deficiencies after bariatric surgery, including hypocalcemia and hypovitaminosis D [14]. Hence, she required a prolonged hospital stay to ensure adequate replacement of vitamin D and calcium, upon her presentation of hypocalcemia post denosumab treatment. Unfortunately, optimal vitamin and mineral replacement post bariatric surgery may have been neglected, or ignored, as she had not had any proper work-up and follow-up following the surgery. Consequently, on the basis of current guidelines, she should have received adequate vitamin supplementations, including vitamin D [15]. Adhering to supplementations should also be stressed upon patients, while highlighting consequences of noncompliance such as hypocalcemia.

## Conclusion

This case demonstrates the risk of profound and refractory hypocalcemia, following the initiation of denosumab in a patient with a prior history of bariatric surgery, in the absence of adequate baseline investigations. We underscore the importance of detecting vitamin and mineral deficiencies prior to denosumab administration. Additionally, this case highlights the need to educate patients about the importance of initiation and adherence to long-term supplementation, post gastric bypass surgery, and continuous follow-up, with regular blood investigations, to prevent complications including osteoporosis and other malnutrition-related illnesses.

## Data Availability

Not applicable.
